# Early-start and conventional-start peritoneal dialysis: a Chinese cohort study on outcome

**DOI:** 10.1080/0886022X.2020.1743310

**Published:** 2020-03-25

**Authors:** Ying Wang, Yang Li, Haiyun Wang, Ying Ma, Danna Ma, Dongli Tian, Bingyan Liu, Zijuan Zhou, Wei Yang, Xuemei Li, Jie Cui, Limeng Chen

**Affiliations:** a Department of Nephrology, Peking Union Medical College Hospital, Chinese Academy of Medical Sciences, Beijing, China; b Nephrology Division, People’s Hospital of Ningxia Hui Autonomous Region, Yinchuan, China; c Nephrology Division, Massachusetts General Hospital, Boston, MA, USA

**Keywords:** Early-start dialysis, peritoneal dialysis, prognosis

## Abstract

**Background:**

Early-start peritoneal dialysis (PD) is an effective option for patients need unplanned dialysis. However, there are few studies on the long-term prognosis of early-start PD patients.

**Methods:**

In this retrospective study, 635 eligible patients from 1 March 1996 to 30 September 2016 were included, and divided into three groups according to the duration of break-in period: 3 days or less, 4–13 days and more than 14 days. Patients started PD within 2 weeks and after 2 weeks were defined as early-start and conventional-start, respectively. The primary outcome was all-cause mortality, and the secondary outcome measures were peritonitis free survival and technical survival. Mechanical and infectious complications in the first 180 days were also analyzed.

**Results:**

Early-start PD patients were more likely to have higher serum total carbon dioxide and creatinine levels and lower serum albumin, Kt/v, creatinine clearance (Ccr) and residual glomerular filtration rate (rGFR) levels at the start of PD. The median follow-up period was 30 months (interquartile range, 13-53 months). A worse survival was observed in the early-start group than that in the conventional-start group (*p* < 0.001), even adjustment for the covariates (HR 1.549, 95%CI 1.104–2.173, *p* = 0.011). In the subgroup analysis, in patients commencing PD after 2006 early-start and conventional-start PD patients had comparable survival. No differences were observed in the rate of infectious and mechanical complications, peritonitis-free survival and technique survival between early-start and conventional-start PD patients.

**Conclusions:**

Early-start PD could be a safe and effective strategy for patients needing unplanned dialysis initiation with the progress of technology on PD.

## Introduction

Chronic kidney disease is a growing global public health problem. Over 900,000 people currently receive maintenance dialysis in Asia [1]. This figure is expected to increase more than twofold between 2010 and 2030, representing the largest absolute growth in patients on dialysis globally [1]. Unplanned dialysis continues to be common in patients with or without nephrology services and in early or late referrals [2–4]. Hemodialysis (HD) is still the first choice for the unplanned dialysis patients, although the permanent access needed to be constructed later [5]. Previous studies have shown comparable patients’ survival and technique survival between PD and HD when started unexpectedly; however, the latter has a higher incidence of dialysis-related complications and bacteremia [6–8]. Given the comparable patients’ survival and less medical costs, early-start PD might be a reasonable alternative for unplanned renal replacement therapy. However, the results of catheter-related complications in early-start PD and conventional-start PD were inconsistent, and long-term technique survival remains lacking [9–12]. The prognosis of early-start PD patients compared with conventional-start patients was reported in few studies with a short follow-up period [13] or incomplete information [2]. The purpose of this study is to compare long-term patient and technique survival between early-start and conventional-start PD patients.

## Materials and methods

### Participants

We performed a longitudinal cohort study at the PD center of Peking Union Medical College Hospital (PUMCH). End stage renal disease (ESRD) patients were included if they were over 18 years old and received regular PD between March 1st, 1996 and September 30th, 2016. Patients with any one of the following criteria were excluded: (1) PD initiation in other hospitals; (2) without regular follow-up after PD initiation (the interval between consecutive two follow-ups >2 months); and (3) incomplete records.

### Early-start PD

Patients were divided into three groups according to the duration of break-in period: 3 days or less (BI ≤3), 4-13 days (BI 4-13) and more than 14 days (BI ≥14). Early-start PD was defined as initiation of PD within 2 weeks after catheter insertion. All Tenckhoff catheter insertions were performed by experienced urologists or nephrologists using open surgery under local anesthesia. The time from placement-to-PD was determined by the nephrologists based on the clinical condition of each patient. An infused volume of 0.8–1.0L in the supine position was used to avoid leakage, which was gradually increased to 2 L per exchange within 3 weeks after catheter insertion if the patients did not present with leakage or abdominal distension. Otherwise, the infused volume would remain at a tolerable level until patients could adapt to a higher volume and gradually titrated to the maximum tolerable volume (less than 2 L). Manual intermittent peritoneal dialysis (IPD) or cycler-assisted dialysis (automated peritoneal dialysis, APD) was used during the first 2 weeks, and continued with continuous ambulatory peritoneal dialysis (CAPD) or APD depending on the clinical situation and preference of patients. All patients were dialyzed using a glucose-based PD solution.

### Data collection

Data for baseline covariates were collected from clinical records. Demographic factors included age, sex, and cause of ESRD. Comorbid diseases included hypertension, diabetes, and existing cardiovascular disease. Dialysis-related variables included PD modalities, peritoneal transport status, dialysis adequacy and residual kidney function (RKF). Dialysis adequacy was estimated by measurement of total weekly Kt/V for urea and total weekly creatinine clearance (Ccr) [14]. Total body water (V) was determined from the Watson formula [15]. RKF was calculated as the average of renal urea and creatinine clearances. A standard peritoneal equilibrium test (PET) performed 6 months after PD initiation was collected. Biochemical covariates included the levels of hemoglobin, serum albumin, total carbon dioxide (TCO2), creatinine, phosphate, calcium, potassium, sodium, uric acid, and intact PTH (iPTH). All laboratory values were measured using automated and standardized methods at a centralized laboratory. Laboratory values collected at the time of initiating PD were considered as baseline values.

### Clinical outcomes

The primary outcome measure was all-cause mortality, and the secondary outcome measures were peritonitis free survival, technical failure, mechanical and infectious complications in the first 180 days. Technique failure was defined as transfer from PD to HD for at least 30 days. Peritonitis free survival was defined as time to first episode of peritonitis. Information on mechanical and infectious complications following PD initiation was collected from clinical records. Mechanical complications included leakage, catheter blockage, and catheter migration. Infectious complications included exit-site infection and peritonitis. Patients were censored at the time of dialysis modality switch, transplantation, transferred to a different renal unit or the end of this study (December 31, 2017) if they were still on PD. The date of death and attributed cause of death were obtained from clinical records for those who died in hospital. For patients who died out of hospital, we interviewed family members by telephone to determine a detailed cause.

The study protocol was approved by the Institutional Review Board (IRB) of PUMCH (IRB approval number s-k734), and all methods were performed in accordance with the relevant guidelines and regulations. All individual information was securely protected and was made available to only the investigators.

### Statistical analysis

The distributional properties of data were expressed as mean ± SD for continuous variables with normal distribution or median (interquartile range) for those with a skewed distribution. Categorical variables were described in terms of their frequency. Variables were compared between early-start and conventional-start groups using Pearson’s chi-squared tests for categorical variables, independent sample t-tests for normally distributed continuous variables and Mann-Whitney rank sum tests for abnormally distributed continuous variables. Patient survival, technique survival, and peritonitis-free survival were evaluated by Kaplan-Meier methods and multivariable Cox proportional hazards survival analyses. The covariates which were significant in univariate analysis or plausibly associated with both exposure and outcome were included in the multivariable Cox proportional hazards survival analysis. A two-sided p-value less than 0.05 was considered to indicate statistical significance. We performed all analyses using SPSS version 20.0 (IBM SPSS statistics, Armonk, New York, USA).

## Results

### Patient characteristics

**Figure 1.**
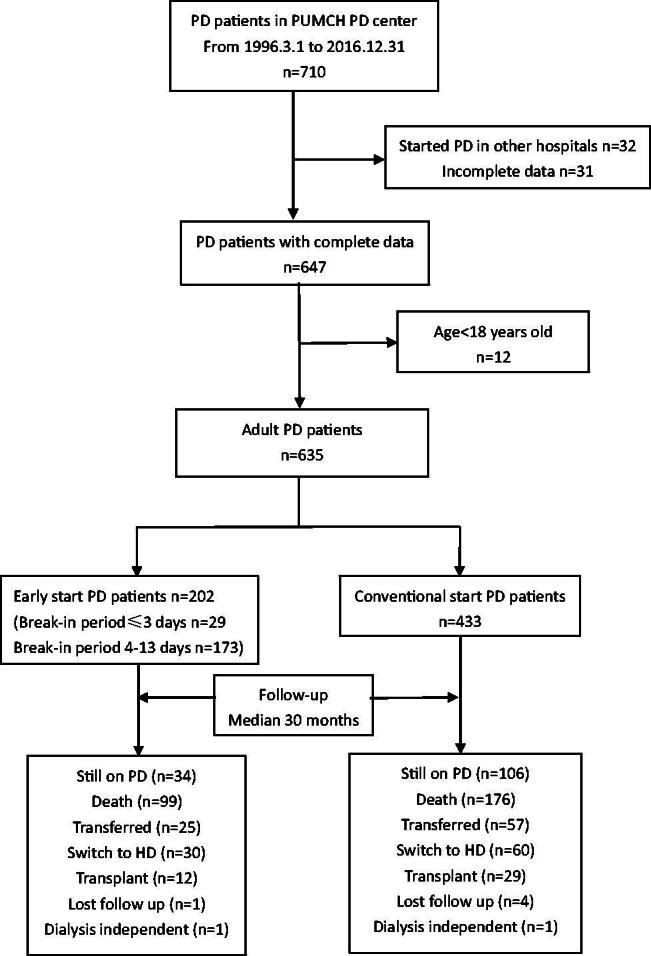
Flow diagram for enrollment and follow-up. PD: peritoneal dialysis; HD: hemodialysis; PUMCH: Peking Union Medical College Hospital.

A total of 635 patients (202 early-start and 433 conventional-start) were included (Figure 1). Baseline characteristics of the study population and patients of different break-in period groups are shown in Table 1. The mean patient age was 59.9 ± 15.3 years old, 49.6% were female. Diabetes was the primary cause of ESRD (36.7%), followed by glomerulonephropathy (25.0%) and hypertension (20.8%). Volume overload was the primary indication for early-start PD in BI ≤3 group (44.8%) and BI 4-13 group (43.9%), followed by uremia (24.1% in BI ≤3 group and 34.1% in BI 4-13 group). Hyperkalemia accounted for 13.8% in BI ≤3 group and 9.2% in BI 4-13 group. No difference was observed in the indication for early-start PD between two groups by Chi-square test (*p* = 0.649).

**Table 1.** Baseline characteristics of patients in different break-in period groups.

		ESPD		CSPD	
	ALL	B*I* ≤ 3 Days	B*I* = 4–13 Days	B*I* ≥ 14 Days	
	*n* = 635	*N* = 29	*N* = 173	*n* = 433	p*-value
Age (years)	59.9 ± 15.3	61.6 ± 18.5	59.3 ± 16.7	60.1 ± 14.5	0.757
Female (%)	315 (49.6)	17 (58.6)	85 (49.1)	213 (49.2)	0.760
Cause of ESRD (%)					0.089
Diabetes	233 (36.7)	9 (31.0)	56 (32.4)	168 (38.8)	
CGN	159 (25.0)	10 (34.5)	51 (29.5)	98 (22.6)	
Hypertension	132 (20.8)	5 (17.2)	41 (23.7)	86 (19.9)	
Others	111 (17.5)	5 (17.2)	25 (14.5)	81 (18.7)	
Comorbidities (%)					
Diabetes	269 (42.4)	10 (34.5)	68 (60.7)	191 (44.1)	0.192
Hypertension	288 (45.4)	10 (34.5)	84 (48.6)	194 (44.8)	0.683
CVD	108 (17.0)	4 (13.8)	26 (15.0)	78 (18.0)	0.323
Time from catheter insertion to PD initiation (days)	14 (13, 18)	1 (0,2)	12 (7,13)	16 (14,19)	<0.001
Chronic PD modalities (%)					0.080
APD	67 (10.6)	2 (6.9)	13 (7.5)	52 (12.0)	
CAPD	568 (89.4)	27 (93.1)	160 (92.5)	381 (88.0)	
Baseline Laboratory results					
Hemoglobin (g/L)	91.5 ± 17.5	97.8 ± 19.6	90.0 ± 17.1	91.7 ± 17.4	0.662
Albumin (g/L)	34.4 ± 6.0	32.4 ± 6.4	33.7 ± 5.8	34.7 ± 6.1	0.034
K (mmol/L)	4.5 ± 0.8	4.5 ± 1.0	4.5 ± 0.8	4.5 ± 0.8	0.826
Na (mmol/L)	138.4 ± 4.1	138.5 ± 5.5	138.9 ± 4.1	138.3 ± 3.9	0.104
TCO_2_ (mmol/L)	24.3 ± 4.7	25.4 ± 6.2	25.1 ± 4.7	24.0 ± 4.6	0.017
Ca (mmol/L)	2.17 ± 0.28	2.23 ± 0.22	2.16 ± 0.29	2.17 ± 0.28	0.917
P (mmol/L)	1.62 ± 0.49	1.77 ± 0.69	1.68 ± 0.48	1.59 ± 0.48	0.056
Creatinine (μmol/L)	732.0 ± 312.7	766.5 ± 444.0	777.5 ± 331.3	712.7 ± 294.4	0.037
Urea (mmol/L)	26.7 ± 11.1	31.6 ± 17.3	27.7 ± 12.0	26.1 ± 10.3	0.083
UA (μmol/L)	455.6 ± 150.2	409.4 ± 167.0	466.9 ± 166.5	454.0 ± 142.8	0.749
PTH (pg/ml)	141.2 (50.1,292.0)	136.0 (79.0, 262.0)	152.7 (39.7,326.5)	142.0 (50.4, 292.0)	0.825
eGFR (ml/min/1.73 m^2^)	6.77 ± 3.68	6.58 ± 3.63	6.32 ± 3.05	6.96 ± 3.89	0.072
Kt/V^a^	2.43 ± 0.68	2.02 ± 0.35	2.26 ± 0.56	2.51 ± 0.71	<0.001
Ccr^a^ (L/week/1.73 m^2^)	79.5 ± 28.3	61.3 ± 13.9	73.6 ± 22.1	82.4 ± 30.0	<0.001
rGFR^a^ (ml/min)	3.17 (1.64,5.11)	1.17 (0.76, 2.61)	2.49 (1.16,4.79)	3.58 (1.90, 5.44)	<0.001
nPCR^a^ (g/kg/day)	0.92 ± 0.27	0.88 ± 0.24	0.90 ± 0.30	0.92 ± 0.26	0.302
Peritoneal transport status^b^ (%)					0.599
High	60 (13.8)	2 (15.4)	12 (11.4)	46 (14.5)	
High average	231 (53.1)	6 (46.2)	59 (56.2)	166 (52.4)	
Low average	132 (30.3)	5 (38.5)	29 (27.6)	98 (30.9)	
Low	12 (2.8)	0 (0)	5 (4.8)	7 (2.2)	
Indication for early-start PD					
Hyperkalemia		4 (13.8)	16 (9.2)		
Uremia		7 (24.1)	59 (34.1)		
Volume overload		13 (44.8)	76 (43.9)		
Others or unavailable		5 (17.2)	22 (12.7)		
PD starting time					0.002
Before 2006	153 (24.1)	12 (41.4)	52 (30.1)	89 (20.6)	
After 2006	482 (75.9)	17 (58.6)	121 (69.9)	344 (79.4)	

Values are expressed as the mean ± SD, number (percentage) or median (i.e. 25th and 75th percentiles). ESPD: early-start peritoneal dialysis; CSPD: conventional-start peritoneal dialysis; BI: break-in period; ESRD: end stage renal disease; CGN: chronic glomerulonephropathy; CVD: cardiovascular disease; APD: automated peritoneal dialysis; CAPD: continuous ambulatory peritoneal dialysis; eGFR: estimated glomerular filtration rate; Ccr: creatinine clearance; rGFR: residual glomerular filtration rate; nPCR: normalized protein catabolic rate.

*ESPD compared with CSPD.

^a^ Kt/V, Ccr, eGFR, and nPCR were evaluated 3 months after PD initiation.

^b^ PET was evaluated 6 months after PD initiation.

Compared with conventional-start patients, early-start patients had lower serum albumin and higher serum TCO2 and creatinine levels at baseline, and no significant difference of the other clinical data present between the two groups. Kt/v, Ccr, and rGFR levels were lower in early-start patients than conventional-start patients 3 months after PD initiation. The proportion of patients who initiated PD before 2006 was higher in early-start PD patients compared with conventional-start PD patients.

### Mechanical and infectious complications

**Figure 2.**
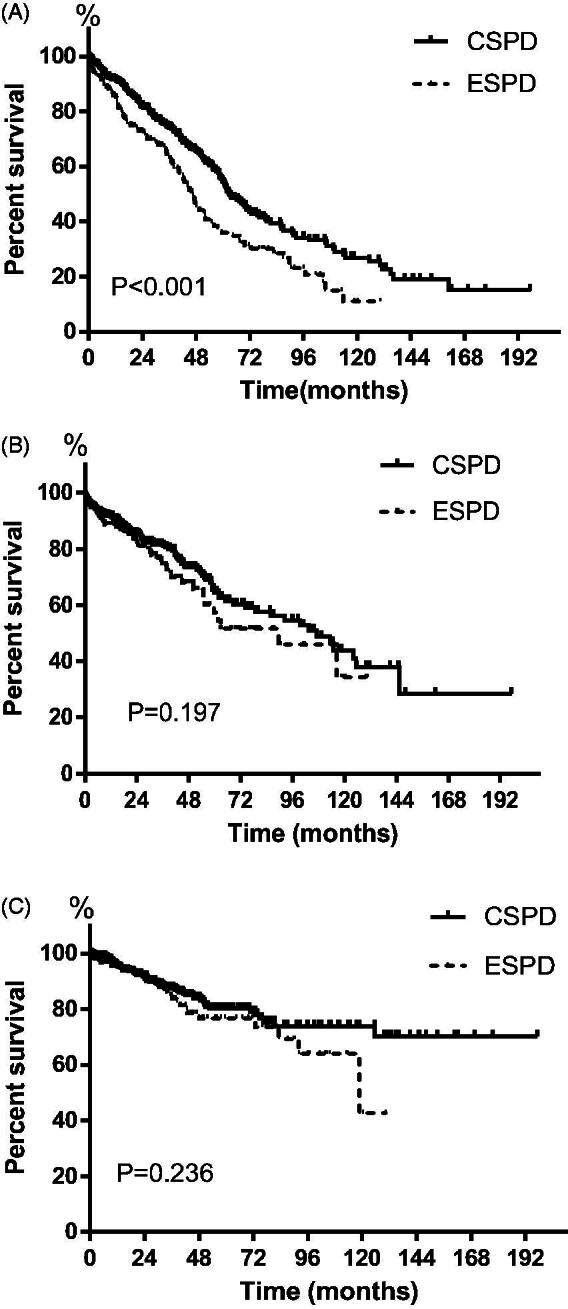
Patient-survival (A), peritonitis-free survival (B) and technique survival (C) for early-start compared with conventional-start in entire patients. CSPD: conventional-start peritoneal dialysis; ESPD: early-start peritoneal dialysis.

In BI ≤ 3 group, the prevalence rate of mechanical complications at 1 month, 3 months and 6 months after PD commencement was 3.4%, 10.3%, and 10.3%, while the relevant infectious complications rate was 6.9%, 6.9%, and 10.3%, respectively. No significant difference was observed in the rate of mechanical and infectious complications between early-start and conventional-start patients at either time point. All pericatheter leakage happened in the first month following catheter insertion. No significant difference was observed between early-start and conventional-start patients in the prevalence rate of all kinds of mechanical and infectious complications at either time point was observed (Table 2). The peritonitis rate in early-start and conventional-start patients was similar (0.19 vs 0.18, episodes per patient-year, *p* = 0.926), consistent with the comparable peritonitis-free survival between the two groups (Figure 2).

**Table 2.** Mechanical and infectious complications after PD commencement in different break-in period groups.

	ESPD		CSPD	p*-value
	B*I* ≤ 3 Days *N* = 29	B*I* = 4–13 Days *N* = 173	B*I* ≥ 14 Days *N* = 433	
1 Month after PD commencement				
Mechanical complications (%)	1 (3.4)	20 (11.6)	49 (11.3)	0.730
Leakage (%)	0 (0)	1 (0.6)	4 (0.9)	0.930
Catheter migration (%)	0 (0)	9 (5.2)	22 (5.1)	0.724
Catheter blockage (%)	1 (3.4)	12 (6.9)	33 (7.6)	0.581
Infectious complications (%)	2 (6.9)	21 (12.1)	51 (11.8)	0.869
Exit-site infection (%)	1 (3.4)	13 (7.5)	38 (8.8)	0.420
Peritonitis (%)	1 (3.4)	8 (4.6)	13 (3.0)	0.357
3 Month after PD commencement				
Mechanical complication (%)	3 (10.3)	35 (20.2)	72 (16.6)	0.516
Catheter migration (%)	0 (0)	14 (8.1)	36 (8.3)	0.536
Catheter blockage (%)	3 (10.3)	24 (13.9)	52 (12.0)	0.645
Infection complication (%)	2 (6.9)	25 (14.5)	61 (14.1)	0.789
Exit-site infection (%)	1 (3.4)	13 (7.5)	38 (8.8)	0.420
Peritonitis (%)	1 (3.4)	12 (6.9)	23 (5.3)	0.578
6 Month after PD commencement				
Mechanical complication (%)	3 (10.3)	39 (22.5)	88 (20.3)	0.915
Catheter migration (%)	0 (0)	14 (8.1)	41 (9.5)	0.282
Catheter blockage (%)	3 (10.3)	28 (16.2)	66 (15.2)	0.993
Infection complication (%)	3 (10.3)	38 (22.0)	99 (22.9)	0.449
Exit-site infection (%)	2 (6.9)	23 (13.3)	73 (16.9)	0.139
Peritonitis (%)	1 (3.4)	16 (9.2)	31 (7.2)	0.589

ESPD: early-start peritoneal dialysis; CSPD: conventional-start peritoneal dialysis; BI: break-in period; PD: peritoneal dialysis.

*ESPD compared with CSPD.

### Technique and patient survival

During a median follow-up period of 30 months (interquartile range, 13-53 months), 275 (43.3%) patients died, 90 (14.2%) switched to HD, 82 (12.9%) were transferred to a different dialysis center, 41 (6.5%) received a kidney transplant. Cardiovascular disease (49.8%) and infection (27.3%) were the two main causes of death.

In BI ≤ 3 group, the 6-month, 1-, 3- and 5-year patient survival rate was 92.4%, 84.0%, 69.5% and 37.9%, while in BI4-13 group it was 91.5%, 86.2%, 61.9% and 35.4%, respectively. The Kaplan-Meier analysis showed overall patient survival of early-start patients was significantly lower than that of conventional-start patients (median survival time 47 months versus 63 months, *p* < 0.001, Figure 2). The 6-month, 1-, 3- and 5-year patient survival rate was 91.6%, 85.9%, 63.0% and 36.1% in early-start patients, and 94.2%, 91.4%, 74.0% and 54.4% in conventional-start patients (*p* < 0.001). Multivariable Cox regression analysis indicated that as well as age, diabetes, and hypoalbuminemia, early-start PD was the independent risk factor for the mortality of PD patients (Table 3). For early-start PD patients, multivariable Cox regression analysis identified age (HR 1.055, 95%CI 1.024–1.087, *p* < 0.001) and diabetes (HR 3.033, 95%CI 1.600–5.753, *p* = 0.001) as independent risk factors for mortality.

**Table 3.** Risk factors for mortality in entire peritoneal dialysis patients.

	Crude hazard ratio		Adjusted hazard ratio	
	Hazard ratio (95%CI)	*p*-value	Hazard ratio^a^ (95%CI)	*p*-value
Female	0.899 (0.709–1.141)	0.381		
Age	1.057 (1.046–1.068)	<0.001*	1.060 (1.045–1.076)	<0.001*
Diabetes	2.439 (1.912–3.112)	<0.001*	2.824 (2.028–3.932)	<0.001*
Hypertension	1.324 (1.043–1.682)	0.021*	0.932 (0.669–1.298)	0.676
CVD	1.797 (1.356–2.381)	<0.001*	1.234 (0.843–1.805)	0.279
ESPD	1.564 (1.220–2.005)	<0.001*	1.549 (1.104–2.173)	0.011*
APD	0.458 (0.290–0.723)	0.001*	0.752 (0.432–1.311)	0.315
HGB^b^ (every 10g/L)	0.901 (0.835–0.973)	0.008	0.934 (0.837–1.048)	0.261
Hypoalbuminemia^c^	1.725 (1.466–2.029)	<0.001*	1.324 (1.059–1.656)	0.014*
eGFR	1.058 (1.033–1.083)	<0.001*	1.027 (0.989–1.067)	0.166
rGFR	1.007 (0.959–1.058)	0.784		
Ccr	1.001 (0.996–1.006)	0.598		
Kt/V	0.910 (0.740–1.120)	0.375		
nPCR	0.419 (0.239–0.732)	0.002*	0.963 (0.492–1.886)	0.913
High peritoneal transport	1.530 (1.021–2.292)	0.040*	1.208 (0.747–1.954)	0.440

CVD: cardiovascular disease; ESPD: early–start peritoneal dialysis; APD: automated peritoneal dialysis; eGFR: estimated glomerular filtration rate; rGFR: residual glomerular filtration rate; Ccr: creatinine clearance rate; nPCR: normalized protein catabolic rate.

^a^ Variables included in the multivariable Cox proportional hazards survival analysis: age, diabetes, hypertension, cardiovascular disease, early-start PD, automated peritoneal dialysis, mean hemoglobin levels in the first 6 months, hypoalbuminemia, eGFR, nPCR, and high peritoneal transport.

^b^ Mean values of the first 6 months after peritoneal dialysis initiation.

^c^ Mean values of the first 6 months divided into 3 degrades：<30g/L, 30–34.9 g/L, and ≥35 g/L. The latter group was used as reference.

*p < 0.05.

**Figure 3.**
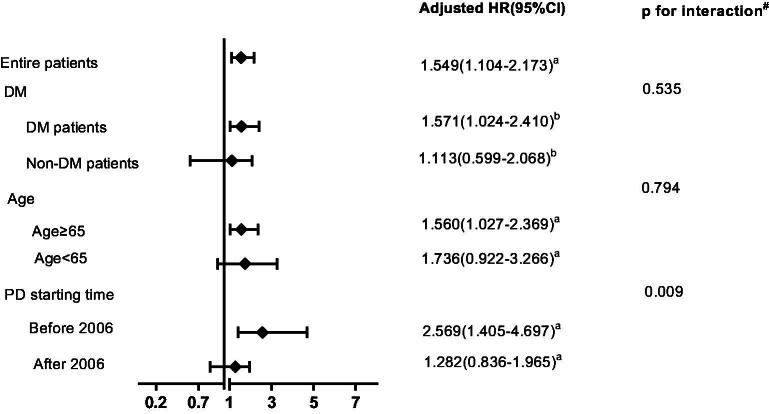
Hazard ratios of early-start peritoneal dialysis on patient survival according to subgroups of diabetes status, age, and PD starting time. ^#^Test for interaction between DM, age and PD starting time with early-start PD on patient survival. DM: diabetes mellitus; PD: peritoneal dialysis. ^a^adjusted for age, diabetes, hypertension, cardiovascular disease, automated peritoneal dialysis, mean hemoglobin levels in the first 6 months, hypoalbuminemia, eGFR, high peritoneal transport and nPCR. ^b^adjusted for age, hypertension, cardiovascular disease, automated peritoneal dialysis, mean hemoglobin levels in the first 6 months, hypoalbuminemia, eGFR, high peritoneal transport and nPCR.

In the subgroup analysis, conventional-start PD still had better survival than early-start PD in patients commencing PD before 1 January 2006 (pre-2005). However, this survival advantage vanished in patients commencing PD after 2006 (post-2005). Complicated with diabetes or older than 65 years did not impact the effect of early-start PD on patient survival (Figure 3).

Ninety patients switched to HD over the study period, including 30 (14.9%) early-start patients and 60 (13.9%) conventional-start patients. The early-start patients had comparable technique survival to conventional-start patients (Figure 2). Cox regression analysis showed that the modality of early-start PD was not the risk factor of technique failure in PD patients (HR 1.304, 95%CI 0.840–2.025, *p* = 0.237). Peritonitis was the main cause of technique failure in early-start PD (36.7%) and conventional-start PD (56.7%). In multivariable Cox regression analysis, young age and higher peritonitis rate were evaluated as independent risk factors for technique failure in early-start patients after adjusting for gender, diabetes, and high or low peritoneal transport (Table 4).

**Table 4.** Risk factors for technique failure in early-start peritoneal dialysis patients.

	Crude hazard ratio		Adjusted hazard ratio^a^	
	Hazard ratio (95%CI)	*p*-value	Hazard ratio (95%CI)	*p*-value
Female	0.676 (0.445–1.026)	0.066	0.524 (0.196–1.402)	0.198
Age	0.986 (0.973–0.999)	0.037*	0.962 (0.934–0.990)	0.009*
DM	0.800 (0.514–1.245)	0.323	0.739 (0.225–2.424)	0.618
HTN	1.082 (0.713–1.641)	0.711		
CVD	0.740 (0.393–1.393)	0.351		
APD	0.865 (0.460–1.627)	0.652		
eGFR	0.930 (0.811–1.067)	0.301		
Ccr	0.996 (0.975–1.017)	0.684		
Kt/V	0.550 (0.212–1.424)	0.218		
High or low peritoneal transport	1.415 (0.405–4.944)	0.587	1.501 (0.409–5.500)	0.540
Peritonitis (episodes/year)	1.954 (0.933–4.095)	0.076	3.461 (1.674–7.155)	0.001*

DM: diabetes; HTN: hypertension; CVD: cardiovascular disease; APD: automated peritoneal dialysis; eGFR: estimated glomerular filtration rate; Ccr: creatinine clearance rate.

^a^ Variables included in the multivariable Cox proportional hazards survival analysis: female, age, diabetes, high or low peritoneal transport and peritonitis.

*p < 0.05.

## Discussion

Our study indicated that conventional-start PD does confer a significant survival advantage compared to early-start PD; however, this survival advantage is lost for patients starting PD post-2005. Except for the survival difference, early-start PD patients had comparable complications and technique survival to that of conventional-start PD patients.

In conventional situation, PD is usually started 14 days after catheter insertion. Urgent PD used to be defined as the use of the PD catheter within 14 days of insertion. However, this definition is considered less satisfactory [16]. It was suggested urgent start PD be reserved for patients with truly urgent presentations requiring PD within 72 h of catheter insertion and PD started between 3 and 14 days after catheter insertion be termed early-start PD. This helps to understand and compare the literature. In this study, we compared the prognosis of patients who started PD within 2 weeks and after 2 weeks. To be more precise, early-start PD was used to define the former group.

The studies focused on the prognosis of early-start PD patients compared with conventional-start PD patients are very few and all of the two studies reported comparable survival rates between the two groups [2,13]. Although Ivansen et al. observed higher mortality in unplanned PD patients than planned patients, the difference vanished after adjusting by age, gender, co-morbidity, serum albumin and time of referral [2]. In our study, the 6-month survival rate of early-start PD patients (91.6%) was better than findings reported by Gorriz *et al.* (82.4%) and Bitencourt *et al.* (89.6%) [17,18], but the long-term overall patient survival of early-start PD patients was inferior to conventional-start PD patients, which was different from the previous studies [2,13]. Only one study observed the similar 2-year survival rate in early-start and conventional-start patients [13]. Their patients were much younger (50.4 years versus 59.9 years) and had lower diabetes rate (27.9% versus 42%) than our patients. Both age and diabetes were independent risk factors for the mortality of PD patients in our study and previous studies [19–21]. However, when we adjusted the age and diabetes in subgroup analysis, these two variables didn’t impact the prognosis of early-start PD. Compared with conventional-start PD patients, the Kt/V, Ccr and rGFR of early-start PD patients were lower, because of the more severe clinical symptoms and lower RKF. In this study rGFR (RKF) at 3 months after PD initiation did not independently predict the mortality as reported before [14]. The possible explanation may be that one-time rGFR especially at the beginning of PD is not as much important as those reported during a period for mortality [22,23]. There is no evidence that higher Kt/V or Ccr were associated with better prognosis, as long as the mean value of Kt/V and Ccr in early-start and conventional-start groups were approximately 1.7 and 50 L/week/1.73 m^2^, respectively [23,24].

In the past two decades, the technical of PD improved so quickly, we further observed the mortality of early-start PD patients in different years. During the first 10 years, conventional-start patients had better survival than that of early-start patients; however, in the following 10 years, the difference disappeared. Although patients starting PD pre-2005 and post-2005 groups had similar levels of serum albumin and rate of primary disease, the older age and lower residual kidney function (RKF) at the start of PD in pre-2005 group were observed. In the past 10 years, great progress in the PD treatment was achieved. Percutaneous PD catheter insertion facilitates timely catheter placement. Automated PD effectively remove uremic toxins, correct electrolyte and volume imbalance, while did not increase in the incidence of early complications during the short break-in period [25]. Therefore, more and more unplanned ESRD patients had the chance to choose PD as an alternative of urgent-start dialysis, with two times of patients in the post-2005 group than that of pre-2005 group. The total patients started PD in the pre-2005 group and post-2005 group was 153 and 482, respectively. On the other hand, larger center and/or higher proportions of PD patients in a dialysis center were reported to be associated with lower peritonitis rates [26], improved technique and patient survival [27,28]. The progress also included more precise guidelines of the International Society for Peritoneal Dialysis (ISPD) and improved understanding of PD treatment. Biocompatible solutions and incremental PD were more and more used to maintain residual kidney function. The team of specialized PD nurses also played a very important role in patients’ education, training, primary care and dealing with the complications of urgent situations expertly.

Our study indicated comparable rates of mechanical complications between early-start and conventional-start PD groups, similar to the results of Ghaffari and Yang’s reports [9,11]. However, previous studies reported the higher rate of mechanical complications in early-start patients than that of conventional-start patients [8,10], especially in patients who commenced PD within 24 h of catheter insertion and surgical replacement of catheters were often needed [8]. Since the immediate commencement and high initial dwell volumes could be possible causes of mechanical complications [29,30], a lower initial dwell and starting PD 48 h after catheter placement were used in our patients which might help to reduce the mechanical complications. We also followed the same pre-operative protocol including the bowel preparing which was believed the important reason for the catheter migration in early-start group [10]. Such progress of technology and management in early-start PD patients contribute to the comparable rate of mechanical complications between the early-start and conventional-start PD groups in our study.

In this study, the long-term infectious complications, peritonitis free survival and technique survival between early-start and conventional-start groups were also similar, which agreed with previous studies [9–12]. However, few studies had followed more than 2-year peritonitis-free and technique survival [12], and our patients were followed more than 10 years. The peritonitis rate in both groups of our patients was 0.18–0.19 episodes per patient-year which was much lower than 0.5 episodes per patient-year required by the ISPD guidelines [31]. In our PD center, the well-designed patient training program is applied for both early-start and conventional-start PD patients by dedicated nurses. The basic training time (ten-hour a day for five consecutive days) and learning approach includes doing through practice, made the patients or their families to be accustomed to the PD operation with fewer infectious complications.

There are several limitations of this study. First, as a retrospective observational study, the early-start on PD decided by the nephrologists and the option of patient, which would lead to the selection bias between the two groups. Secondly, the study represents the experience of a single center and limits the generalizability of the results. Thirdly, the timing for nephrology referral was not included in this study which could be associated with dialysis modality choice and timing for dialysis initiation.

## Conclusion

We first concluded that compared with conventional-start PD patients, early-start PD patients had similar rates of catheter-related complications, technique, peritonitis-free survivals and all-cause mortality in the past 10 years. Early-start PD could be a safe and effective strategy for patients needing unplanned dialysis initiation with the progress of technology on PD.
